# Education and Communication in Veterinary Clinical Practice

**DOI:** 10.3390/ani14172622

**Published:** 2024-09-09

**Authors:** Paul C. Mills

**Affiliations:** School of Veterinary Science, The University of Queensland, Gatton Campus, Gatton, QLD 4343, Australia; p.mills@uq.edu.au

Effective communication is a hallmark of successful veterinary clinical practice. Communication underpins professionalism, establishes effective teamwork, and is essential to veterinary–client relationships. Teaching or developing effective communication is an increasingly important component of the ‘hidden’ curriculum for veterinary educators, introduced and reinforced throughout the teaching program. Effective communication is also a focus for prospective employers when considering a new graduate. Successful communication must also consider the rapidly changing modalities of how people communicate and, importantly, how important communication will be toward resilience in a highly stressful profession.

The ‘intervention’ of COVID-19 rapidly taught everyone that traditional approaches to communication were not always feasible or safe. A paradigm shift occurred for many higher education providers, particularly during the peak stages of the pandemic, when face-to-face (F-2-F) teaching was either not permitted or impractical. Indeed, the emergency response teaching [1] largely consisted of rapid, large-scale online adaptation of teaching and assessment strategies [2]. eLearning became the new paradigm as teachers and educators attempted to maintain student learning within pandemic restrictions that varied by country and region. Blended learning and flipped classrooms have been reported to be educationally sound [3,4,5,6], so it was pleasing to include examples in this Special Issue where veterinary educators have also used these approaches successfully. Using flipped classrooms and/or blended learning to successfully teach cardiac auscultation and anatomical structure are examples of teaching that would normally only be considered effective in an F-2-F setting. An important outcome of using flipped classrooms is that students are more likely to engage in deep learning strategies due to a reduction in cognitive load [7,8].

It was interesting that several articles in this Special Issue focused on enhancing interpersonal and interprofessional communication skills. Returning to the impact of COVID-19, it was highlighted that human physicians tend to consider veterinarians as having a superior knowledge and understanding of zoonoses [9,10]. This superior comprehension and ability to diagnose [11] places veterinarians in a unique position at the human–animal interface [12], elevating the importance of interprofessional communication.

An important underlying theme of this Special Issue is that successful and safe graduate transition to veterinary clinical practice is associated with communication competency development [13,14]. It was therefore pleasing to see veterinary educators describing their success in using blended learning approaches to improve communication and establish personal relationships between veterinarians and the pet owners, which has been reported to improve the quality and outcomes of medical treatments [15,16]. This Special Issue takes this further by teaching students to self-assess their communication skills, adding an important cognitive alignment to the increasingly popular use of self-directed learning (SDL).

The importance of effective communication exceeds many barriers that could normally be expected to restrict learning. What the papers included in this Special Issue have in common is veterinary educators adopting and adapting aspects of learning theory to enhance the outcomes in veterinary science students by focusing on communication at multiple levels of the curriculum.
